# Aortic endograft infection after thoracic endovascular aortic repair: two case reports and literature review

**DOI:** 10.3389/fcvm.2025.1549613

**Published:** 2025-02-25

**Authors:** Danping Peng, Guang Xu, Bin Wang, Xin Li, Yang Wang

**Affiliations:** Center of Infectious Disease and Pathogen Biology, Department of Infectious Diseases, The First Hospital of Jilin University, Changchun, China

**Keywords:** TEVAR, complications, long-term management, aortic endograft infection, aortoesophageal fistula, gastrointestinal bleeding

## Abstract

With the maturity of thoracic endovascular aortic repair (TEVAR) technology and its increasing application in clinical practice, complications and long-term management after TEVAR have become issues of concern. Here, we report two cases of TEVAR for thoracic aortic dissection. One patient developed recurrent fever 6 years after TEVAR and underwent multiple courses of antibiotic therapy with a poor response. He came to our hospital 6 months later and presented with gastrointestinal bleeding. Imaging revealed the presence of an aortic abscess around the stent graft involving the esophagus and mediastinum. The patient's condition deteriorated rapidly after admission, and he ultimately succumbed to hemorrhagic shock. Another patient developed recurrent fever 1 year after surgery. Imaging studies suggested an aortic abscess with involvement of the esophagus, and the patient chose conservative treatment. After long-term anti-infective treatment, the infected lesions remained but had decreased in size. Aortic endograft infection complicated by multiple organ involvement is a rare complication of TEVAR and has a high mortality rate. After an extended postsurgical period, patients who have undergone TEVAR often lack regular follow-up and are easily overlooked. Our cases highlight the importance of early prevention, early diagnosis, and appropriate management of late complications following TEVAR.

## Introduction

Diseases of the aorta, including thoracic aortic aneurysm (TAA) and type B aortic dissection (TBAD), have become life-threatening emergencies in some acute complex conditions. With the development of endovascular techniques, surgical treatment for thoracic aortic emergencies has shifted from traditional open surgery to minimally invasive thoracic endovascular aortic repair (TEVAR). TEVAR was recommended by the European Society for Vascular Surgery (ESVS) as a first-line treatment for descending aortic rupture, penetrating aortic ulcers, intramural hematoma, blunt traumatic aortic injury, and complicated acute type B aortic dissection in 2017 (1). Although TEVAR has achieved impressive therapeutic effects and has successfully saved the lives of countless patients, complications related to stent placement should not be overlooked. Here, we report two cases of patients who developed severe complications 1 and 6 years after TEVAR. Both of these patients developed an aortic abscess around the stent graft involving the esophagus, and ultimately, one of the patients died. Currently, sufficient academic literature on the long-term outcomes of TEVAR treatment are lacking. Our case report aims to provide a reference for the prevention and diagnosis of long-term complications after TEVAR.

## Case report 1

A 59-year-old male patient underwent TEVAR surgery 6 years prior due to thoracic aortic dissection. Postoperatively, he regularly took metoprolol and irbesartan to control his blood pressure, and his condition remained stable. Six months prior, the patient developed irregular fever without apparent cause, with a maximum temperature of 39.5°C. He also experienced profuse sweating and fatigue. At a local hospital, chest CT and echocardiography showed no abnormalities. Cephalosporins and penicillins were administered for more than 20 days, resulting in the relief of fever symptoms. However, fever recurred after discharge, and subsequent antibiotic treatment at a local clinic showed poor efficacy. One month prior, the patient developed cough with the expectoration of small amounts of white sputum but did not receive standardized treatment. Five days prior, he experienced increased urinary frequency, urgency, and dysuria. On the morning of admission, the patient experienced hematemesis, characterized by bright red blood in small amounts. He presented to our hospital for evaluation. Over the past 6 months, the cause of his fever has remained unknown, and he has lost 10 kg in weight.

At admission, his physical examination results were as follows: poor general condition, blood pressure, 108/70 mmHg; temperature, 36.4°C; heart rate, 98 beats/min; and respiratory rate, 20 breaths/min. He exhibited epigastric tenderness without abdominal tension or rebound tenderness. Other vital signs are unremarkable.

The laboratory test results were as follows: white blood cell count, 20.5 × 10^9^/L (normal range: 3.5–9.5); neutrophil percentage (NE%), 0.88 (normal range: 0.40–0.75); absolute neutrophil count, 18.1 × 10^9^/L (normal range: 1.8–6.3); and hemoglobin, 112 (normal range: 130–175) g/L. His inflammatory markers were elevated: C-reactive protein was 256.93 (normal range: 0–10) mg/L, procalcitonin was 24.73 (normal range: 0–0.5) ng/ml, the erythrocyte sedimentation rate was 43 (normal range: 0–15) mm/h. blood culture (aerobic + anaerobic) revealed *Streptococcus constellatus* (anaerobic).

**Figure 1:**
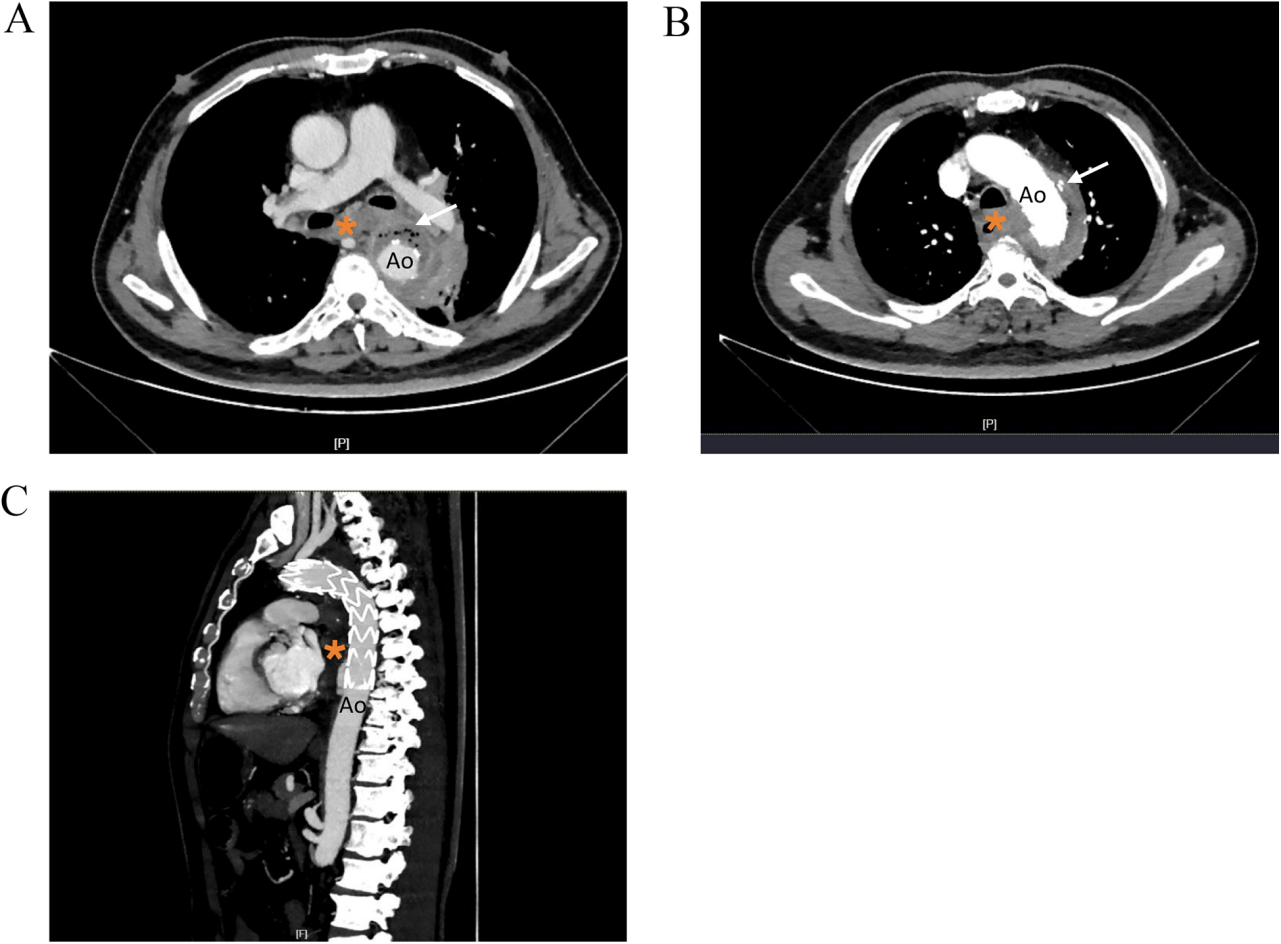
Thoracic aortic CTA images showing. **(A)** Low density shadow and scattered gas shadow around the aortic stent graf. **(B)** Nodular contrast imaging was observed outside the stent. **(C)** Sagittal reformatted images. Ao, descending thoracic aorta; *, esophagus.

Although the patient's chest CT and echocardiogram at other hospitals showed no abnormalities, an infection related to TEVAR could not be excluded. After admission, antibiotic therapy was adjusted to meropenem [1.0, ter in die, intravenous drip (TID, iv. D)] combined with tigecycline [0.05, bis in die, intravenous drip (BID, iv. D)] for infection control. Eight hours after admission, the patient experienced dizziness and passed 400 ml of bloody stools. Emergency endoscopy revealed massive gastric hemorrhage, however, the bleeding source could not be identified, and the procedure was terminated. Subsequent thoracic aortic computed tomography angiography (CTA) suggested postthoracic aortic stent grafting, with thrombosis around the stent and the formation of a local fistula. There were low-density shadows and scattered gas shadows outside the stent, suggesting possible abscess formation. The boundary between the stent and the esophagus and lung tissues was unclear, with haziness in the mediastinal fat gap and a slight accumulation of air. There was also enlargement of the mediastinal and left hilar lymph nodes (Figure 1). Considering the patient's history of stent implantation and clinical presentation, the possibility of periaortic infection combined aortoesophageal fistula, gastrointestinal bleeding, endoleak and mediastinal abscess were considered. Oral intake was prohibited, and omeprazole and octreotide were administered continuously.

Following a comprehensive evaluation by the cardiac surgery team, repeat stenting or surgical intervention was deemed not feasible. Twenty-four hours after admission, the patient's blood pressure progressively decreased to 87/51 mmHg, and his hemoglobin decreased to 66 g/L. Continuous infusion of adrenaline was initiated, and aggressive fluid resuscitation and blood transfusion were performed. However, the patient experienced recurrent hematemesis and melena. Eventually, he died of hemorrhagic shock.

## Case report 2

A 69-year-old male patient underwent TEVAR surgery 1 year prior due to aortic dissection. Postoperatively, he regularly took amlodipine besylate to control his blood pressure. Additionally, he had a 10-year history of diabetes with poor glycemic control. Six months prior, the patient developed fever without apparent cause, with a maximum temperature of 39.5°C and chills, and blood culture revealed Streptococcus intermedius. He was diagnosed with sepsis at our hospital and was discharged after anti-infective treatment. Two months prior, fever and chills recurred, and the patient was admitted to our hospital. CTA of the thoracic aorta revealed a mass lesion around the stent in the lower thoracic aorta with air accumulation. Aortic endograft infection was considered, and the patient chose conservative treatment. He was discharged after anti-infective treatment with cefotidine. Seven days prior, the patient developed fever with cough and expectoration again. Lung CT at our hospital revealed aggravated bilateral pneumonia, bilateral pleural effusion and a lower esophageal mass. He presented to our hospital for further evaluation.

On admission, his physical examination results were as follows: anemic appearance; blood pressure, 152/78 mmHg; and body temperature, 36.4°C. His heart rate was 78 beats/min, and his respiration rate was 20 beats/min.

The laboratory findings were as follows: percentage of neutrophils (NE%), 0.81 (normal range: 0.40–0.75), absolute neutrophil count, 7.35 × 10^9^/L (normal range: 1.8–6.3); and hemoglobin level, 70 (normal range: 130–175) g/L. His inflammatory marker levels were elevated: C-reactive protein, 69.68 (normal range: 0–10) mg/L and erythrocyte sedimentation rate, 80 (normal range: 0–15) mm/h. Blood cultures were negative.

**Figure 2:**
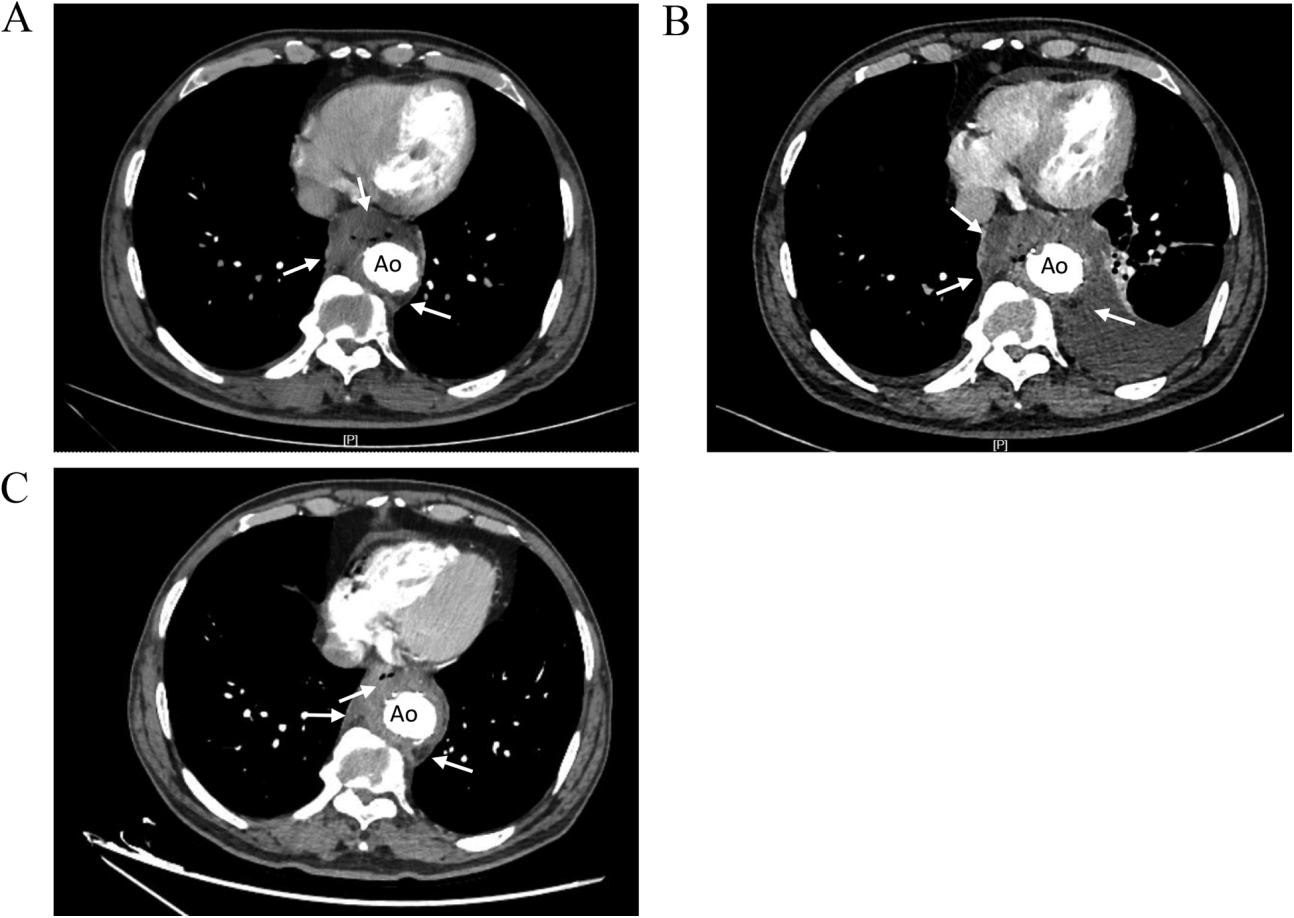
Comparison of thoracic aortic CTA images showing. **(A)** CTA images 2 months before admission. Low-density shadow was found around the stent in the lower thoracic aorta (right side, right anterior, left posterior), and the boundary with the esophagus was not clear. **(B)** CTA images during hospitalization. The low-density shadow around the stent in the lower thoracic aorta had unclear boundary with the esophagus, accompanied by air in it, and the scope of the lesion was larger than before. **(C)** CTA images at 3 months after discharge. The mass lesion around the lower thoracic aortic stent with air accumulation in it, the range of infection was smaller than before. Ao, descending thoracic aorta.

The patient had recurrent fever for 6 months, After the second admission, and CTA still revealed a mass lesion around the stent. Aortic endograft infection was considered. piperacillin-tazobactam [4.5, ter in die, intravenous drip (TID, iv. D)] and tigecycline [0.05, bis in die, intravenous drip (BID, iv. D)] were given for anti-infective treatment. CTA imaging of the thoracic aorta revealed that after thoracic aortic stenting, there was a mass lesion around the lower thoracic aortic stent and the esophageal area with air accumulation. Compared with the previous CTA of the thoracic aorta, the lesion was enlarged in the right anterior area and slightly reduced in the left posterior area, with bilateral pleural effusions and partial atelectasis in the adjacent lower lobe of the left lung. After anti-infective treatment, the patient had no fever, and the infection index decreased. After multidisciplinary consultation with the Departments of Cardiac Surgery, Vascular Surgery, and Infectious Disease, it was considered that the patient had an aortic graft infection involving the esophagus and that surgical treatment was needed; however, the risk of surgery was high, and the patient chose to continue conservative treatment. Minocycline [0.1, bis in die, per os (BID, p.o.)] and amoxicillin [0.5, ter in die, per os (TID, p.o.)] were recommended after discharge. The patient returned to the hospital for reexamination 3 months after discharge, and there was no fever or other symptoms of discomfort. CTA of the thoracic aorta revealed a mass lesion around the stent in the lower thoracic aorta and the esophageal area with air accumulation, although it was significantly smaller than that previously observed (Figure 2).

## Discussion

We report two elderly male patients who developed graft infection following TEVAR. two of our patients developed aortic graft infection involving multiple organs 6 years and 1 year after TEVAR, which is extremely rare and concerning.

The incidence of aortic diseases is increasing annually, and TEVAR technology is increasingly being applied in clinical practice (2). According to a recent publication based on data from the International Registry of Aortic Dissection (IRAD), from 1996 to 2022, the proportion of acute TBAD patients receiving endovascular management increased from 19.1% to 37.2% (3). Clinical studies have shown that compared to open aortic repair, TEVAR results in shorter hospital stays and fewer complications (4). Many patients with aortic diseases significantly benefit from this surgical approach. With the increasing use of endovascular aneurysm repair (EVAR), related complications have also increased (5). Some complications after TEVAR, such as endoleak, paraplegia, and stroke, have been widely reported, and some rare complications include aortoesophageal fistula, endograft collapse, and cerebrovascular events (6–8). Although their incidence is low, they often develop in a malignant manner, posing a threat to the patient's life and requiring close attention. Table 1 summarizes some complications of TEVAR, along with their diagnosis and treatment.

**Table 1:** Complications, clinical presentations, and treatment after TEVAR.

Complications	Clinical manifestation	Treatment	References
Endoleaks	Imaging reveals persistent blood flow perfusion within the residual aneurysmal sac.	Surgical re-intervention can be performed based on the classification of endoleaks, such as endovascular extension grafts, coil embolization, and conversion to open surgery.	(2, 9, 10)
Stent migration	Displacement of the endoprosthesis from its original position by more than 10 mm is observed or any amount of migration resulting in symptoms.	Using an aortic extension cuff or deploying a large balloon to expand the stent can enhance the fixation of the endoprosthesis to the native aortic wall.	(11, 12)
Endograft collapse	Symptoms suggestive of acute aortic occlusion may arise, with imaging revealing stenosis within the graft.	Balloon dilation can be performed, and open aortic repair may be necessary if needed.	(7, 13)
Spinal cord ischemia	Manifests as permanent or transient spinal cord injury.	Preoperative spinal drainage, perioperative epidural corticosteroid use, and spinal drainage.	(14, 15)
Cerebrovascularevents	Some patients present with cerebral ischemia or asymptomatic cerebral infarction.	Follow the standard stroke management guidelines.	(16, 17)
Post-implantation syndrome	Non-infectious persistent fever and elevated inflammatory markers.	Monitor and manage symptomatically; antibiotics are not required.	(18)
Endograft infection	It often presents with fever, back pain, and elevated inflammatory markers. Computed tomography scans show bubbles or fluid around the aortic graft.	Aggressive antimicrobial therapy; Surgical removal of infected endograft.	(19)
Aortoesophageal fistula	Fever, fatigue, occasionally accompanied by sepsis, clear visualization of fistula on endoscopy.	Aggressive antimicrobial therapy, surgical treatment typically involves a combination of aortic procedures (TEVAR, graft replacement, or repair) and esophageal procedures (esophagectomy, esophageal stenting, or repair).	(20, 21)

In our study, the patients experienced recurrent fever, elevated inflammatory marker levels, and an evident aortic abscess around the stent combined with aortic-esophageal fistula. According to the diagnostic criteria for aortic graft infection (AGI) established by the Management of Aortic Graft Infection Collaboration (MAGIC) in the UK (22), the patient received a definite diagnosis of post-TEVAR AGI. AGI is a rare complication of TEVAR, and its diagnosis and management are highly complex. The clinical presentations are diverse, which is unfavorable for disease diagnosis and treatment.

AGI has diverse causes, and based on the time of occurrence, AGI can be classified into early (<4 months) and late (≥4 months) infections (23). Early AGI is typically caused by contamination during EVAR surgery or a preexisting bacterial infection present during EVAR (24). Late AGI can be influenced by a wide range of factors, and any adjacent or distant infection from various sites can be a causative or contributing factor to AGI. For example, bacteremia resulting from urinary tract or respiratory tract infections can lead to bacterial dissemination via the bloodstream to the area around the graft, causing infection (25). Mechanical erosion due to stent displacement, repetitive frictional tears, and intramural fixation hooks can also lead to fistula formation and inflammation (26). Additionally, researchers have found that factors such as the use of immunosuppressive agents by the host or the presence of other conditions, such as malignancies or diabetes, are significant risk factors for AGI (25, 27). In case report 2, the patient had diabetes mellitus and usually had poor glycemic control, which may have increased his risk for infection. Both patients had pulmonary infections during the course of their disease, which also raises the possibility of bacteremia secondary to a respiratory tract infection.

AGI poses catastrophic hazards to patients, as the progression of infection may involve other organs, including the esophagus, trachea, lungs, and mediastinum, leading to complications such as aortoesophageal fistula, aortobronchial fistula, and complex systemic infections (28). The fundamental principles of managing AGI include removing the infected device, performing vascular reconstruction, and administering adjunctive antimicrobial therapy (19). However, vascular grafts are not designed for easy removal, and the surgical procedure imposes significant trauma on patients. One study showed that the 30-day mortality rates for endovascular and open reintervention for failed TEVAR were 6.7% and 15%, respectively (29). For patients who are not candidates for surgery, such as those with a high anticipated surgical mortality, those whose grafts are situated in a location that cannot be resected, or those who decline surgical intervention, antibiotic therapy may present a more favorable alternative to surgical resection. Relevant studies have found that some patients who are inoperable and treated with antibiotics alone have achieved favorable treatment outcomes through long-term, aggressive targeted antibiotic therapy (30, 31). Early targeted antibiotic treatment plays a significant role in eradicating pathogenic microorganisms and reducing the formation of biofilms, among other important effects (30). However, studies have also shown that conservative treatment is difficult to prevent the progression of this disease, and the mortality rate is extremely high (19, 25). One patient in our study was treated conservatively, and the infection lesion was reduced after active anti-infective treatment; however, the infection lesion remained. We will continue to follow this patient.

The long-term management of patients after TEVAR surgery should be given careful attention. Our patient developed graft infection 6 years after stent implantation, and the onset of infection was extremely insidious. By the time the etiology was definitively determined, the condition had become difficult to control. According to the guidelines for postendovascular repair imaging surveillance, imaging examinations should be conducted at 30 days, 6 months, and 12 months postsurgery (32). If no complications are found, imaging should be performed annually thereafter. In clinical practice, vigilance should be maintained for the possibility of complications when patients exhibit symptoms such as fever and pain after stent placement, and laboratory and imaging examinations should be promptly performed. For patients who have already developed complications, interdisciplinary consultations should be sought, and the most appropriate clinical treatment plan should be devised for the patient.

In summary, the therapeutic prospects of TEVAR for aortic diseases are promising, but this procedure also poses significant safety concerns. Enhancing the understanding of rare complications following TEVAR, monitoring the long-term outcomes of patients treated with TEVAR, and focusing on the prevention and timely diagnosis of post-TEVAR complications are of paramount importance.

## Data Availability

The original contributions presented in the study are included in the article/Supplementary Material, further inquiries can be directed to the corresponding author.
